# Supplementary material for: Hydrogen peroxide is required for light-induced stomatal opening across different plant species
Source: Nat Commun. 2024 Jun 14;15:5081. doi: 10.1038/s41467-024-49377-9 (PMC11178795; doi:10.1038/s41467-024-49377-9)
Supplement: Supplementary file 1 — Supplementary Information [file 41467_2024_49377_MOESM1_ESM.pdf]

Supplementary information

Hydrogen Peroxide is Required for Light-Induced Stomatal Opening across Different Plant Species

Shi et al

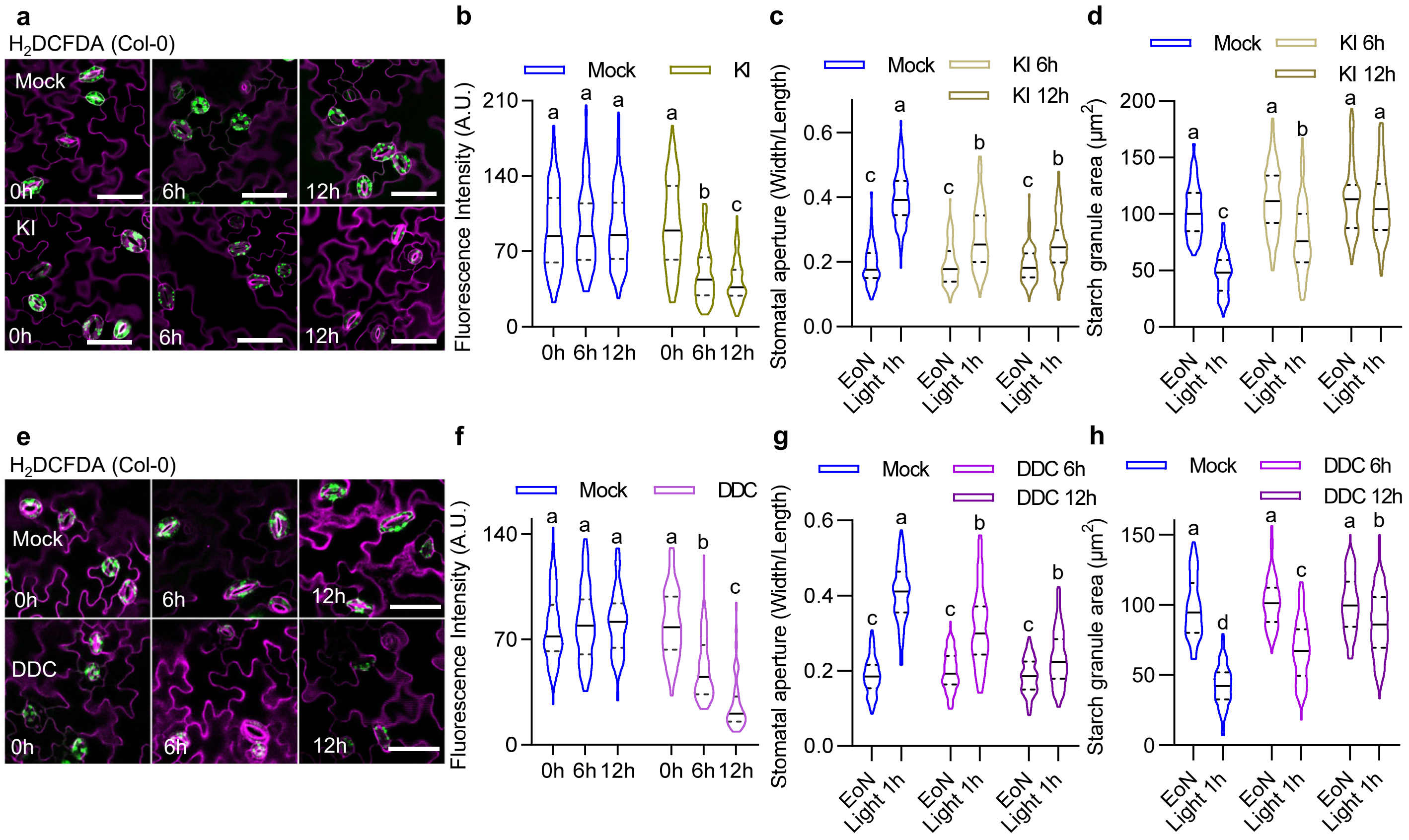

Supplementary Fig. 1 Reduction of H_2O_2 by KI or DDC treatment suppresses the light-induced stomatal opening. **a-d** Measurement of H_2O_2 using H_2DCFDA in guard cells (**a, b**), quantification of stomatal apertures (**c**) and guard cell starch granules (**d**) in guard cells of wild type cotyledon after potassium iodide (KI) treatment. Seedlings of Col-0 were grown on 1/2 MS medium under a 12 h light/12 h dark photoperiod with a 100 μM m^{-2} s^{-1} light intensity for 10 days, and then transferred to the medium containing mock solution or 1 mM KI for different times before observation harvesting plants. **e-h** Measurement of H_2O_2 in guard cells using H_2DCFDA (e, f), 1uantification of stomatal apertures (**g**) and guard cell starch granules (**h**) of wild type cotyledon with or without Diethyldithiocarbamic acid (DDC) treatment. Seedlings of Col-0 were grown on 1/2 MS medium under a 12 h light/12 h photoperiod with a 100 μM m^{-2} s^{-1} light intensity for 10 days, and then transferred to the medium containing mock solution or 1 mM DDC for different times before observation and harvesting plants. H_2DCFDA signaling intensity from at least 100 guard cells of 8 different plants were analyzed using ImageJ software. Scale bars represents 50 μm. The starch granules area and the ratio of stomatal aperture width to length from more than 100 guard cells of at least 12 different plants were measured using ImageJ software. EoN means the end of night, and Light 1h means the white light illumination for 1 hour after the end of night. Different letters above the bars indicate statistically significant differences between samples (Two-way ANOVA analysis followed by Tukey's multiple comparisons test, $p < 0.05$). The solid lines of violin plots in this figure represent median, the dashed lines represent first or third quartile.

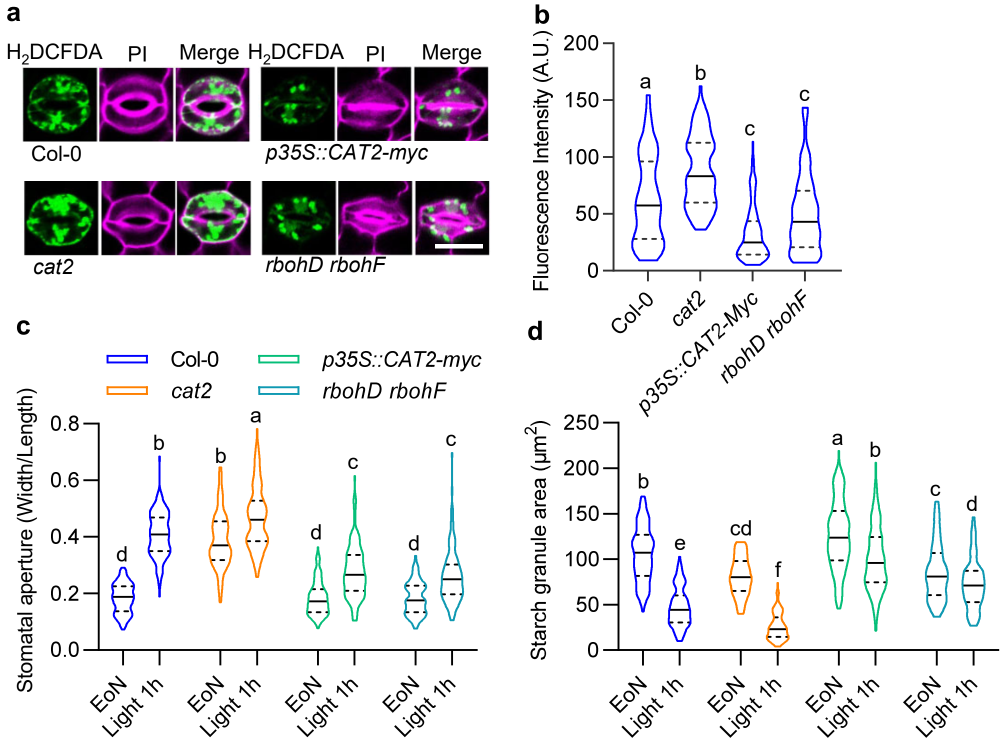

Supplementary Fig. 2 Specific accumulated H_2O_2 in guard cell is required for light induced stomata opening in cotyledon of Arabidopsis. a, b Measurement of H_2O_2 in guard cells of Col-0, *cat2*, *p35S::CAT2-myc*, *rbohD rbohF* cotyledon using H_2DCFDA. Seedlings were grown on 1/2 MS medium under a 12 h light/12 h photoperiod with a 100 μM m^{-2} s^{-1} light intensity for 4 days. H_2DCFDA signaling intensity in at least 100 guard cells from 8 different plants were analyzed by ImageJ software. Scale bars represent 20 μm. Different letters above the bars indicate statistically significant differences between samples (One-way ANOVA analysis followed by uncorrected Fisher's LSD multiple comparisons test, $p < 0.05$). **c, d** Quantification of stomatal apertures (**c**) and guard cell starch granules (**d**) in cotyledons of Col-0, *cat2*, *p35S::CAT2-myc* and *rbohD rbohF* plants. Seedlings were grown on 1/2 MS medium under a 12 h light/12 h dark photoperiod with a 100 μM m^{-2} s^{-1} light intensity for 10 days. The starch granules area and the ratio of stomatal aperture width to length from more than 100 guard cells of at least 12 different plants were measured using ImageJ software. EoN means the end of night, and Light 1 h means the white light illumination for 1 hour after the end of night. Different letters above the bars indicate statistically significant differences between samples (Two-way ANOVA analysis followed by Tukey's multiple comparisons test, $p < 0.05$). The solid lines of violin plots in this figure represent median, the dashed lines represent first or third quartile.

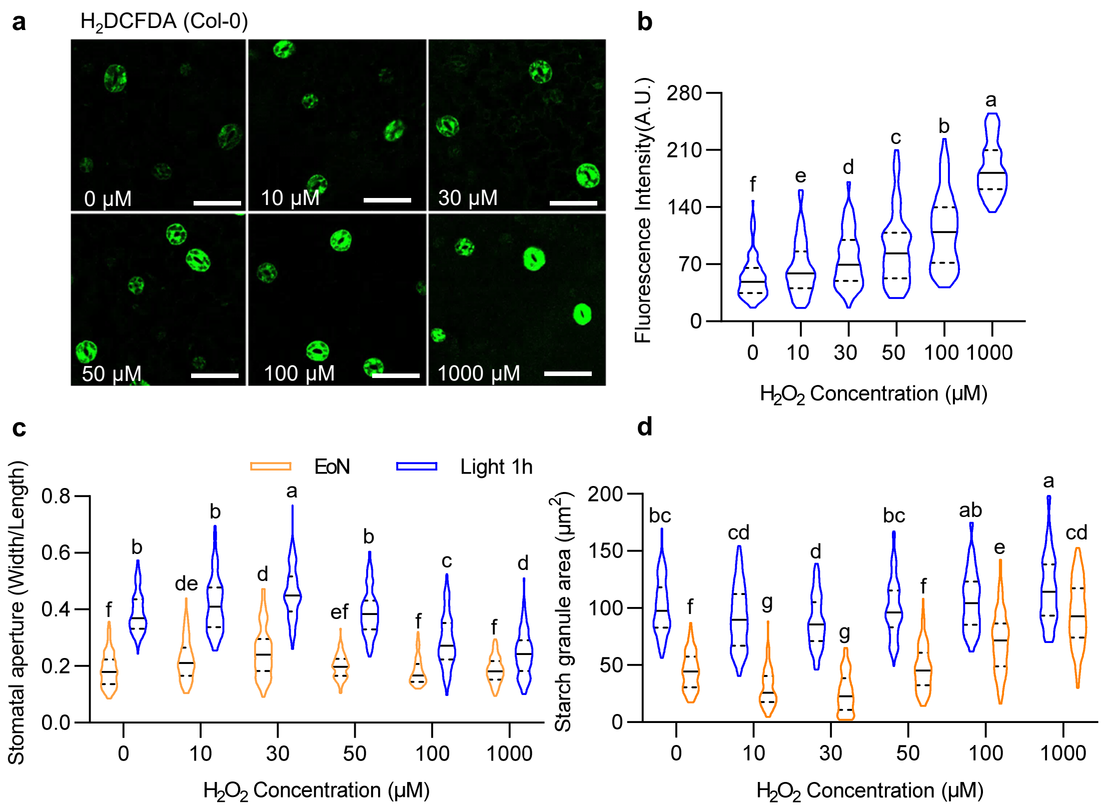

Supplementary Fig. 3 H_2O_2 promotes stomatal opening at low concentrations, but inhibits stomatal opening at high concentrations. a, b Measurement of H_2O_2 in the guard cells of wild type cotyledon treatment with different concentrations of H_2O_2 using H_2DCFDA. Seedlings of Col-0 were grown on 1/2 MS medium under a 12 h light/12 h dark photoperiod with a 100 μM m^{-2} s^{-1} light intensity for 4 days, transferred to the medium containing mock solution or different concentrations of H_2O_2 to grow for 2 h before observation. H_2DCFDA signaling intensity was analyzed in at least 100 guard cells from 8 different plants were analyzed using ImageJ software. Scale bars represents 50 μm. Different letters above the bars indicate statistically significant differences between samples (One-way ANOVA analysis followed by Uncorrected Fisher's LSD multiple comparisons test, $p < 0.05$). **c, d** Quantification of stomatal apertures (**c**) and guard cell starch granules (**d**) in Col-0 plants after different concentration of H_2O_2 treatment. Seedlings of Col-0 were grown on 1/2 MS medium under a 12 h light/12 h dark photoperiod with the 100 μM m^{-2} s^{-1} light intensity for 10 days, transferred to the medium containing mock solution or different concentration of H_2O_2 to grow for 2 h before the end of night, and then harvested at the indicated time points. The starch granules area and the ratio of stomatal aperture width to length from more than 100 guard cells of at least 12 different plants were measured using ImageJ software. EoN means the end of night, and Light 1h means the white light illumination for 1 hour after the end of night. The solid lines of violin plots represent median, the dashed lines represent first or third quartile. Different letters above the bars indicate statistically significant differences between samples (Two-way ANOVA analysis followed by Tukey's multiple comparisons test, $p < 0.05$).

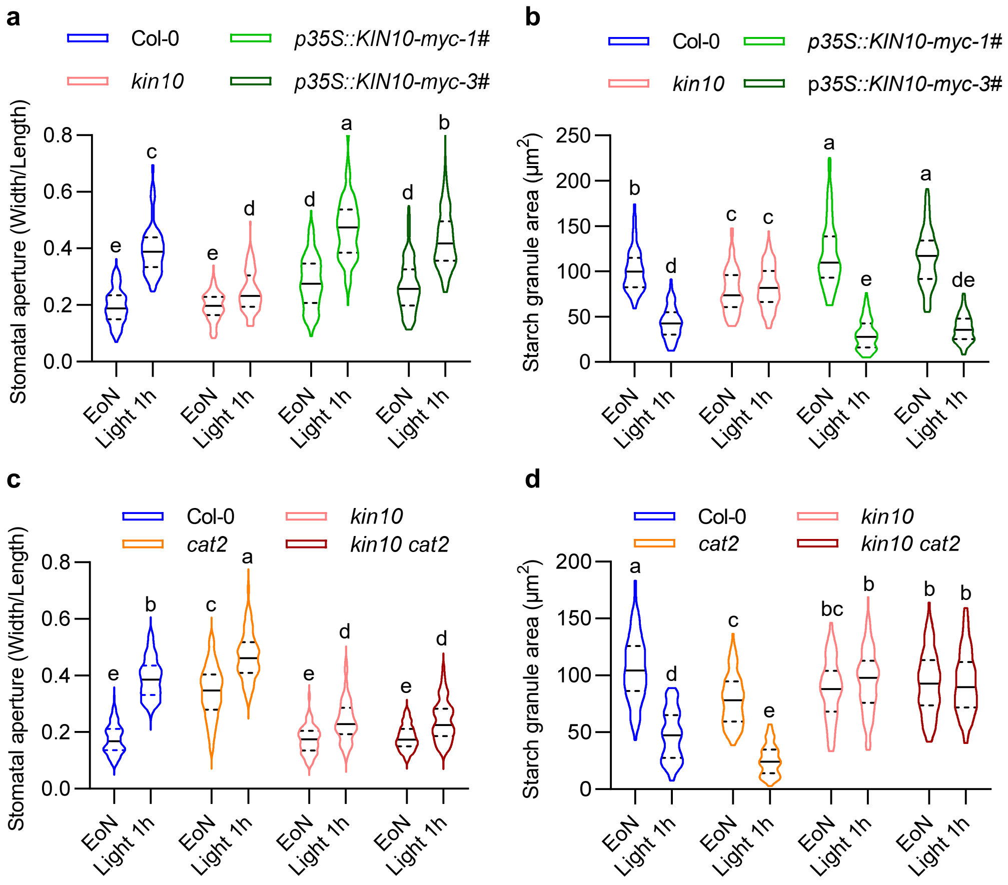

Supplementary Fig. 4 KIN10 is required for H_2O_2 promoted light-induced stomatal opening in cotyledon. a, b Quantification of stomatal apertures (**a**) and guard cell starch granules (**b**) in cotyledons of Col-0, *kin10* and *p35S::KIN10-myc* plants. **c, d** Quantification of stomatal apertures (**c**) and guard cell starch granules (**d**) in cotyledons of Col-0, *kin10*, *cat2* and *kin10 cat2* plants. Seedlings were grown on 1/2 MS medium under a 12 h light/12 h dark photoperiod with the 100 μM m^{-2} s^{-1} light intensity for 10 days. The starch granules area and the ratio of stomatal aperture width to length from more than 100 guard cells of at least 12 different plants were measured using ImageJ software. EoN means the end of night, and Light 1 h means the white light illumination for 1 hour after the end of night. The solid lines of violin plots represent median, the dashed lines represent first or third quartile. Different letters above the bars indicate statistically significant differences between samples (Two-way ANOVA analysis followed by Tukey's multiple comparisons test, $p < 0.05$).

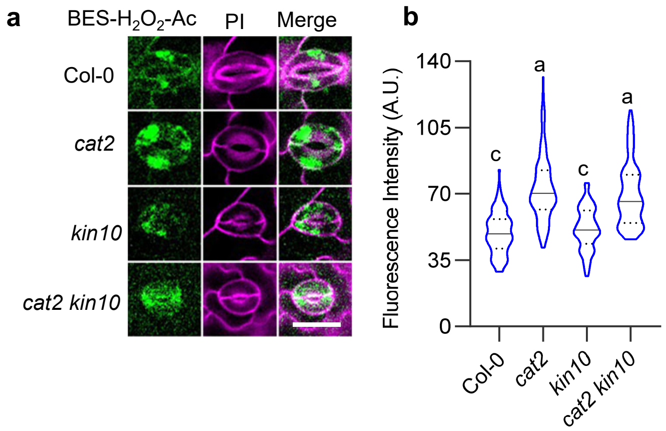

Supplementary Fig. 5 KIN10 does not interfere H_2O_2 accumulates in the guard cells under normal condition. a, b BES-H_2O_2-Ac staining for H_2O_2 in guard cells on the rosette leaves of four-weeks-old Col-0, *cat2*, *kin10*, and *cat2 kin10* plants. These plants were grown on 1/2 MS medium under a 12 h light/12 h dark photoperiod with the 100 µM m^{-2} s^{-1} light intensity for 28 days. Fluorescent signals were taken from more than 100 guard cells in rosette leaves of 10 different plants and analyzed using ImageJ software. Scale bars in confocal images represent 20 µm. The solid lines of violin plots represent median, the dashed lines represent first or third quartile. Different letters above the bars indicate statistically significant differences between samples (One-way ANOVA analysis followed by Tukey's multiple comparisons test, $p < 0.05$).

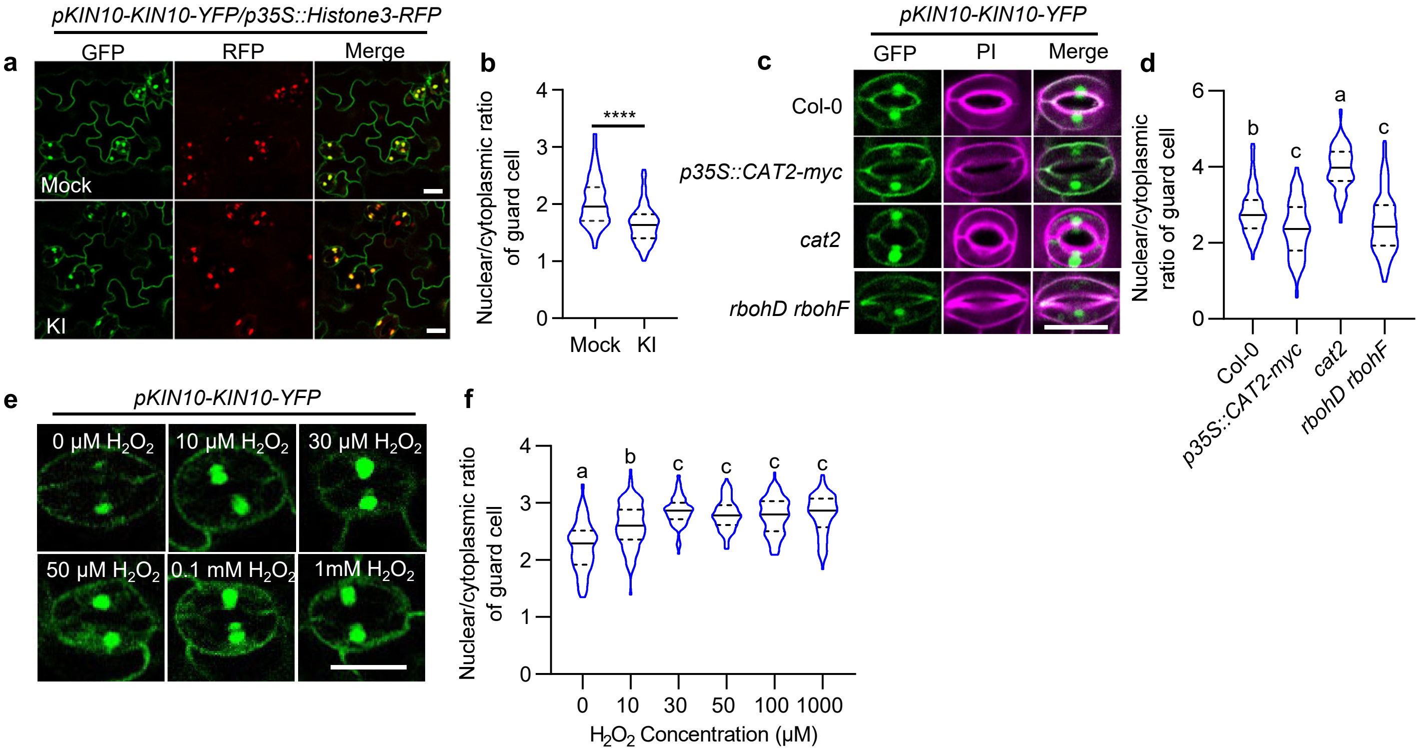

Supplementary Fig. 6 H_2O_2 promotes nuclear localization of KIN10 in guard cells. a, b KI treatment inhibits nuclear localization of KIN10. Seedlings of *pKIN10::KIN10-YFP/p35S::Histone3-RFP* were grown on 1/2 MS medium under a 12 h light/12 h dark photoperiod with the 100 μM m^{-2} s^{-1} light intensity for 10 days and then treated with or without 1 mM KI for 12 hours. The ratio of nuclear and cytoplasmic KIN10-YFP signal intensity from at least 100 guard cells of 8 different plants were analyzed by ImageJ software. Scale bars represents 50 μm. Asterisk indicated statistically significant differences between samples (Student's t test, ****p<0.0001). **c, d** Measurement of ratio of nuclear and cytoplasmic KIN10-YFP in Col-0, *p35S::CAT2-myc*, *cat2* and *rbohD rbohF* plants. Seedlings of *pKIN10::KIN10-YFP,* the plants of *pKIN10::KIN10-YFP* crossed with *p35S::CAT2-myc*, *cat2* and *rbohD rbohF* mutants were grown on 1/2 MS medium under a 12 h light/12 h dark photoperiod with the 100 μM m^{-2} s^{-1} light intensity for 8 days. The ratio of nuclear and cytoplasmic KIN10-YFP signal intensity from at least 100 guard cells of different background plants were analyzed by ImageJ software. Scale bars represents 20 μm. Different letters above the bars indicate statistically significant differences between samples (One-way ANOVA analysis followed by Uncorrected Fisher's LSD multiple comparisons test, $p < 0.05$). **e, f** H_2O_2 induces the nuclear localization of KIN10 in guard cells of plants. Seedlings of *pKIN10::KIN10-YFP* were grown on 1/2 MS medium under a 12 h light/12 h dark photoperiod with the 100 μM m^{-2} s^{-1} light intensity for 10 days and then treated with or without different concentrations of H_2O_2 for 6 hours. The ratio of KIN10-YFP nuclear and cytoplasmic signal intensity from at least 100 guard cells of 10 different plants were analyzed by ImageJ software. Scale bars represents 20 μm. Different letters above the bars indicate statistically significant differences between samples (One-way ANOVA analysis followed by Uncorrected Fisher's LSD multiple comparisons test, $p < 0.05$). The solid lines of violin plots in this figure represent median, the dashed lines represent first or third quartile.

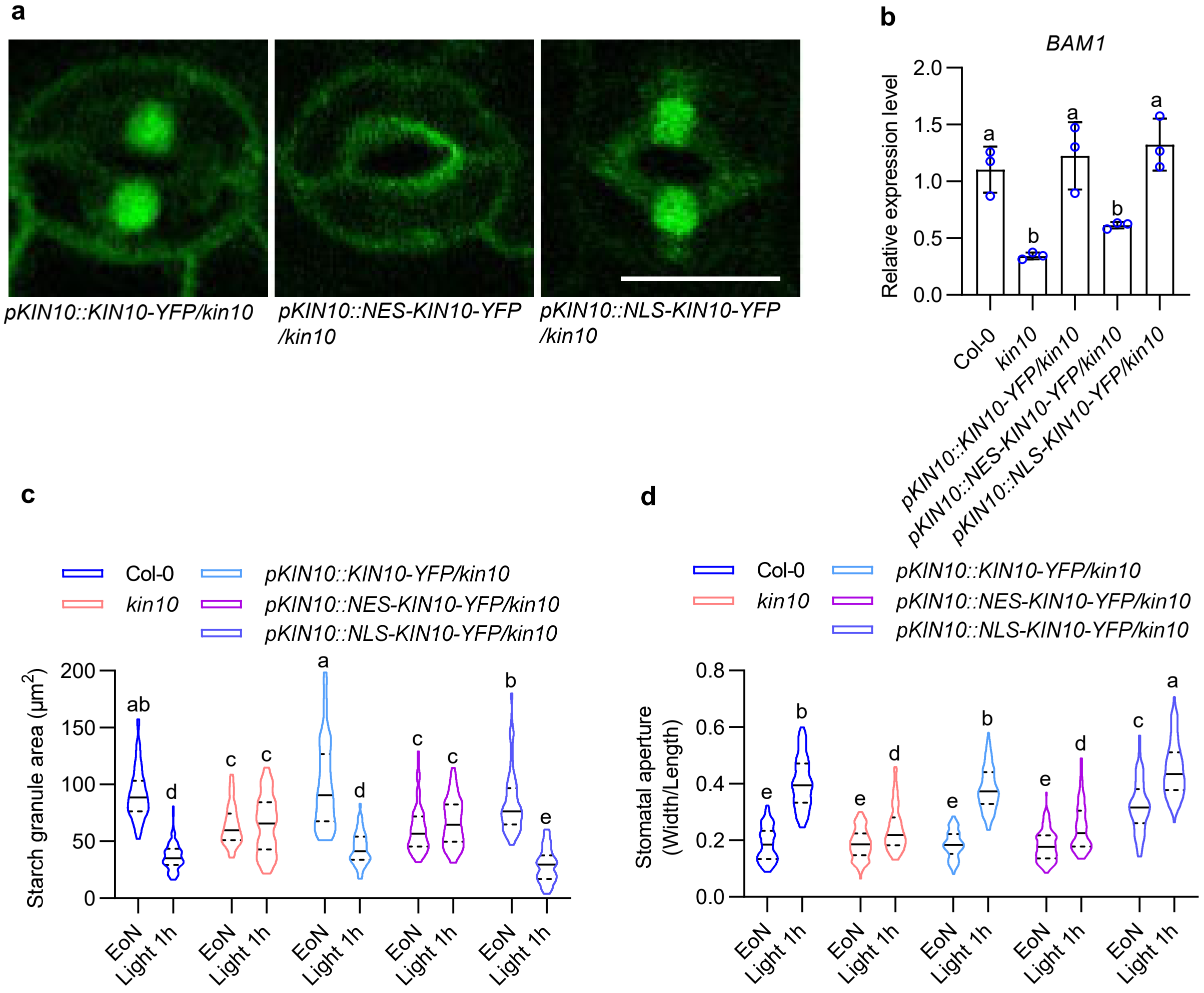

Supplementary Fig. 7 The nuclear localized KIN10 is essential for the light-induced stomatal opening. a Subcellular localization of KIN10 protein in guard cell of *pKIN10::KIN10-YFP/kin10, pKIN10::NES-KIN10-YFP/kin10* and *pKIN10::NLS-KIN10-YFP /kin10* transgenic plants. Scale bars represents 20 µm. **b**, qRT-PCR analysis of the expression of *BAM1* in Col-0, *kin10*, *pKIN10::KIN10-YFP/kin10, pKIN10::NES-KIN10-YFP/kin10* and *pKIN10::NLS-KIN10-YFP/kin10* plants. Plants of Col-0, *kin10*, *pKIN10::KIN10-YFP/kin10, pKIN10::NES-KIN10-YFP /kin10* and *pKIN10::NLS-KIN10-YFP /kin10* were grown 1/2 MS medium under a 12 h light/12 h dark photoperiod with a 100 µM m^{-2} s^{-1} light intensity for 28 days. Stomata from rosette leaves were enriched thought Stomata Tape-Peel method. Error bars indicate standard deviation (S.D.). Different letters above the bars indicate statistically significant differences between samples (One-way ANOVA analysis followed by uncorrected Fisher's LSD multiple comparisons test, $p < 0.05$). **c, d** Quantification of guard cell starch granules (**c**) and stomatal apertures (**d**) in Col-0, *kin10*, *pKIN10::KIN10-YFP/kin10, pKIN10::NES-KIN10-YFP/kin10* and *pKIN10::NLS-KIN10-YFP/kin10* plants. Seedlings were grown on 1/2 MS medium under a 12 h light/12 h dark photoperiod with the 100 µM m^{-2} s^{-1} light intensity for 10 days. The starch granules area and the ratio of stomatal aperture width to length from more than100 guard cells of at least 12 different plants were measured using ImageJ software. EoN means the end of night, and Light 1 h means the white light illumination for 1 hour after the end of night. Different letters above the bars indicate statistically significant differences between samples (Two-way ANOVA analysis followed by Tukey's multiple comparisons test, $p < 0.05$). The solid lines of violin plots in this figure represent median, the dashed lines represent first or third quartile.

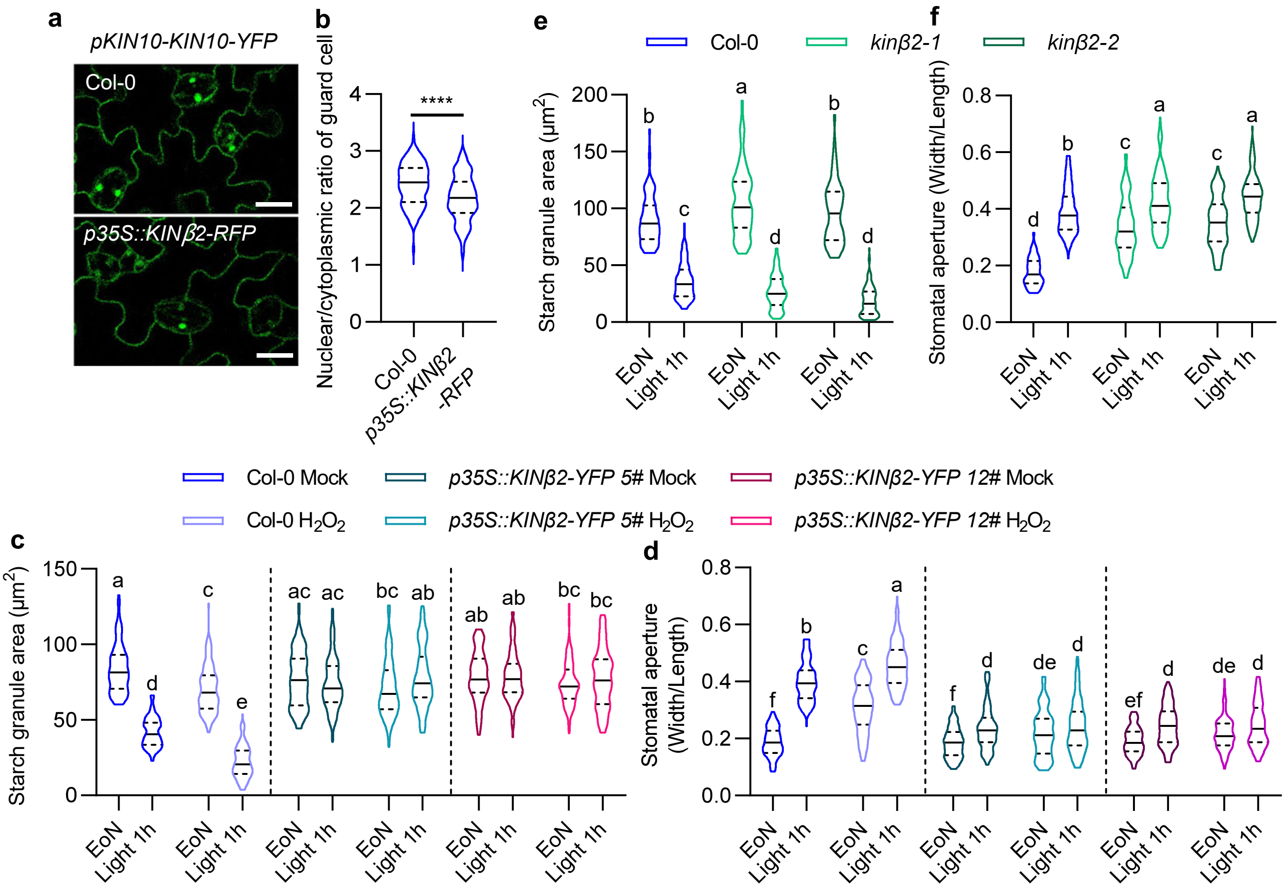

Supplementary Fig. 8 KINβ2 represses light-induced stomatal opening. a, b KINβ2 restrict KIN10 protein nuclear localization in guard cell. *pKIN10::KIN10-YFP* and *pKIN10::KIN10-YFP/p35S::KINβ2-RFP* plants were grown on 1/2 MS medium under a 12 h light/12 h dark photoperiod with the 100 μM m^{-2} s^{-1} light intensity for 10 days. The ratio of KIN10-YFP nuclear and cytoplasmic signal intensity from at least 100 guard cells of 8 different plants were analyzed using ImageJ software. Scale bars represents 20 μm. Asterisk indicated statistically significant differences between the samples (Student's t test, ****p<0.0001). **c, d** Quantification of guard cell starch granules (**c**) and stomatal apertures (**d**) in Col-0 and *p35S::KINβ2-YFP* plants with or without H_2O_2. Seedlings were grown on 1/2 MS medium under a 12 h light/12 h dark photoperiod with a 100 μM m^{-2} s^{-1} light intensity for 10 days, then transferred to the medium with or without 30 μM of H_2O_2 to grow for 2 h before the end of night, and then harvested at the indicated time points. **e, f** Quantification of guard cell starch granules (**e**) and stomatal apertures (**f**) in Col-0, *kinβ2-1* and *kinβ2-2* plants. Seedlings of these plants were grown on 1/2 MS medium under a 12 h light/12 h dark photoperiod with the 100 μM m^{-2} s^{-1} light intensity for 10 days. The starch granules area and the ratio of stomatal aperture width to length from more than100 guard cells of at least 12 different plants were measured using ImageJ software. EoN means the end of night, and Light 1 h means the white light illumination for 1 hour after the end of night. Different letters above the bars indicate statistically significant differences between samples (Two-way ANOVA analysis followed by Tukey's multiple comparisons test, $p < 0.05$). The solid lines of violin plots in this figure represent median, the dashed lines represent first or third quartile.

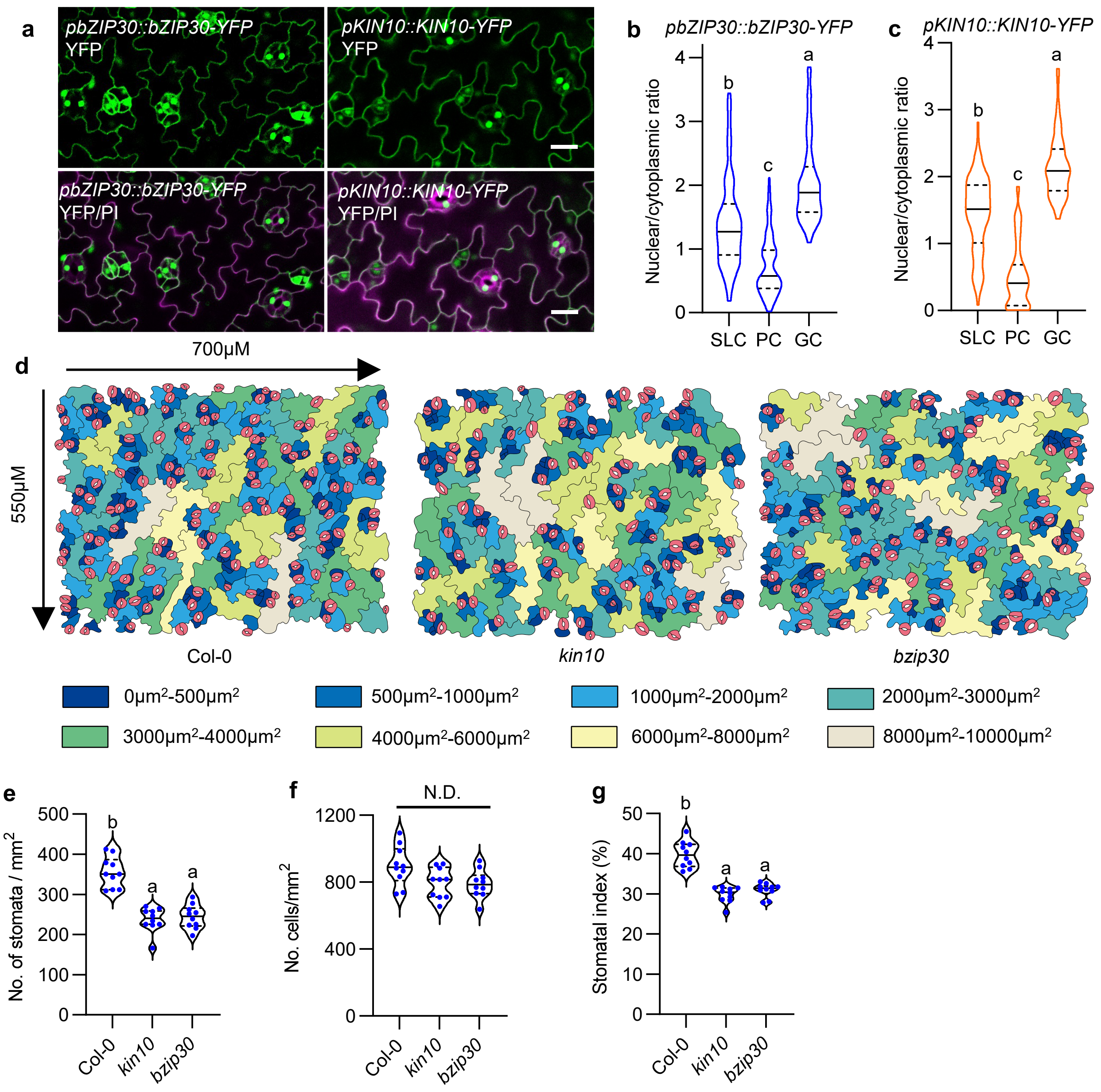

Supplementary Fig. 9 bZIP30 and KIN10 possesses similar protein localization pattern in Arabidopsis epidermis and promotes stomatal development. a-c, The subcellular locations of *pbZIP30::bZIP30-YFP* and *pKIN10::KIN10-YFP* are similar in Arabidopsis cotyledon epidermal cells. Seedlings of *pbZIP30::bZIP30-YFP* and *pKIN10::KIN10-YFP* were grown in 1/2 MS medium under a 12 h light/12 h dark photoperiod with the 100 μM m^{-2} s^{-1} light intensity for 4 days. bZIP30-YFP and KIN10-YFP are in green, PI-marked cell outlines are in purple. Scale bars in confocal images represent 20 μm. Scatter plots showed bZIP30-YFP (**b**) and KIN10-YFP (**c**) exhibit similar nuclear-to-cytoplasmic ratio in different types of epidermal cells. “SLC” indicates stomatal lineage cells, “PC” indicates pavement cells, “GC” indicates guard cells. **d-g,** Abaxial epidermis of cotyledons of Col-0, *kin10* and *bzip30.* (**d**). The seedlings of Col-0, *kin10* and *bzip30* plants were grown on 1/2 MS liquid medium with 1% sucrose under a 12 h light/12 h dark photoperiod with the 100 μM m^{-2} s^{-1} light intensity for 10 days. Cell size distribution is presented as a color scale. Stomata density (**e**), total epidermal cells density (**f**) and stomatal index (**g**) of 10-day-old Col-0, *kin10* and *bzip30* plants were quantified. Different letters above the bars indicate statistically significant differences between samples (One-way ANOVA analysis followed by uncorrected Fisher’s LSD multiple comparisons test, $p < 0.05$). The solid lines of violin plots in this figure represent median, the dashed lines represent first or third quartile.

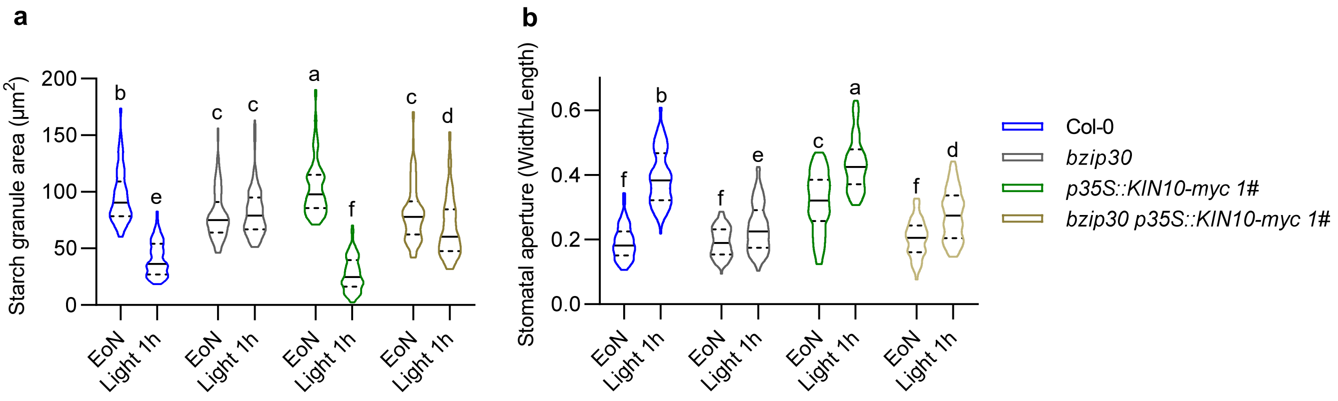

Supplementary Fig. 10 bZIP30 is required for KIN10 promoted light-induced stomatal opening in cotyledon. a, b Quantification of guard cell starch granules (**a**) and stomatal apertures (**b**) in cotyledon of Col-0, *bzip30*, *p35S::KIN10-myc 1#* and *bzip30 p35S::KIN10-myc 1#* plants. Seedlings of Col-0, *bzip30*, *p35S::KIN10-myc 1#* and *bzip30 p35S::KIN10-myc 1#* plants were grown on 1/2 MS medium under a 12 h light/12 h dark photoperiod with the 100 μM m^{-2} s^{-1} light intensity for 10 days. The solid lines of violin plots in this figure represent median, the dashed lines represent first or third quartile. Different letters above the bars indicate statistically significant differences between samples (Two-way analysis ANOVA followed by Tukey's multiple comparisons test, $p < 0.05$).

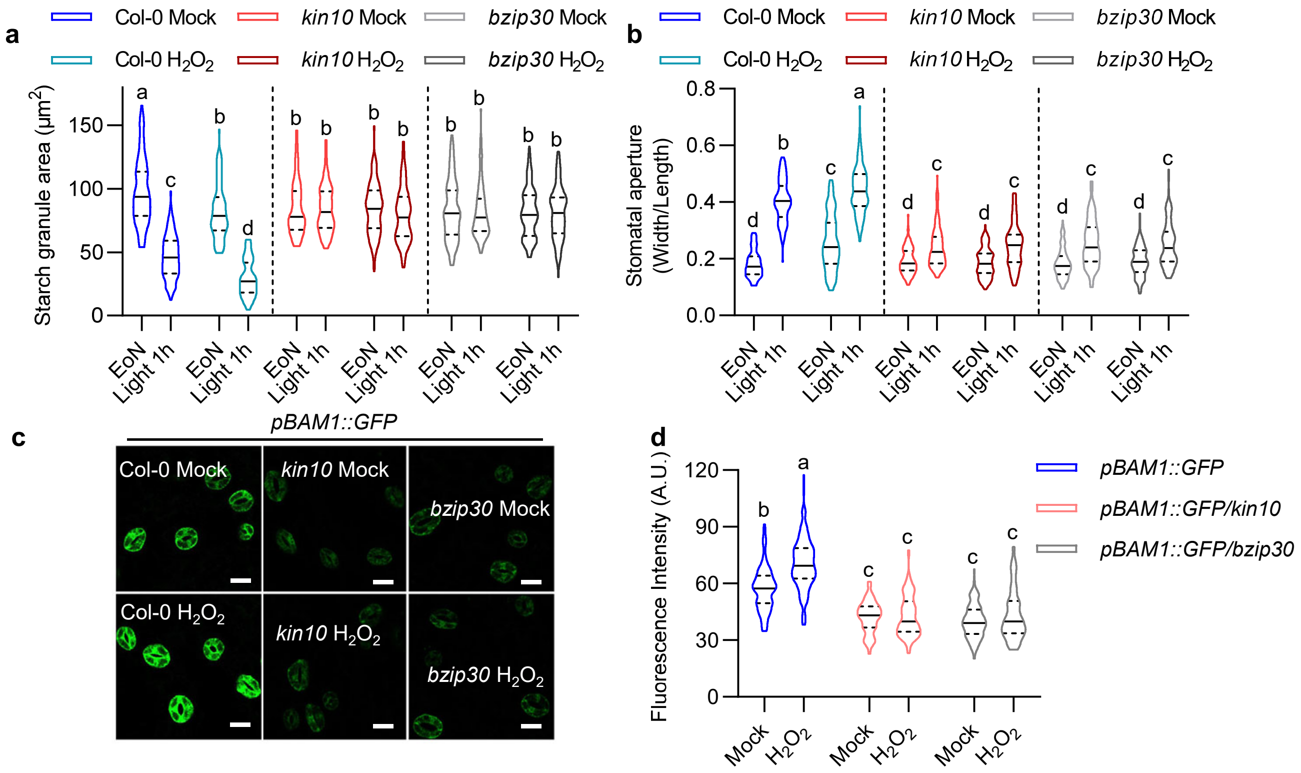

Supplementary Fig. 11 KIN10 and bZIP30 is required for low concentration of H_2O_2-mediated promotion of light-induced stomatal opening. a, b Quantification of guard cell starch granules (**a**) and stomatal apertures (**b**) in Col-0, *kin10* and *bzip30* plants with or without H_2O_2. Seedlings were grown on 1/2 MS medium under a 12 h light/12 h dark photoperiod with the 100 µM m^{-2} s^{-1} light intensity for 10 days, then transferred to the medium with or without 30 µM of H_2O_2 to grow for 2 h before the end of night, and then harvested at the indicated time points. The starch granules area and the ratio of stomatal aperture width to length from more than100 guard cells of at least 12 different plants were measured using ImageJ software. EoN means the end of night, and Light 1 h means the white light illumination for 1 hour after the end of night. Different letters above the bars indicate statistically significant differences between samples (Two-way ANOVA analysis followed by Tukey's multiple comparisons test, $p < 0.05$). **c, d** Mutations of KIN10 and bZIP30 inhibit H_2O_2 induced *BAM1* expression. Seedling of *pBAM1::GFP*, *pBAM1::GFP/kin10* and *pBAM1::GFP/bzip30* were grown on 1/2 MS medium under 12 h light/12h dark photoperiod with the 100 µM m^{-2} s^{-1} light intensity for 10 days, and then treated with or without 30 µM of H_2O_2 for 3 hours. GFP signal from more than 100 guard cells of at least 10 different plants were analyzed using ImageJ software. Scale bars represent 20 µm. Different letters above the bars indicate statistically significant differences between samples (Two-way ANOVA analysis followed by Tukey's multiple comparisons test, $p < 0.05$). The solid lines of violin plots in this figure represent median, the dashed lines represent first or third quartile.

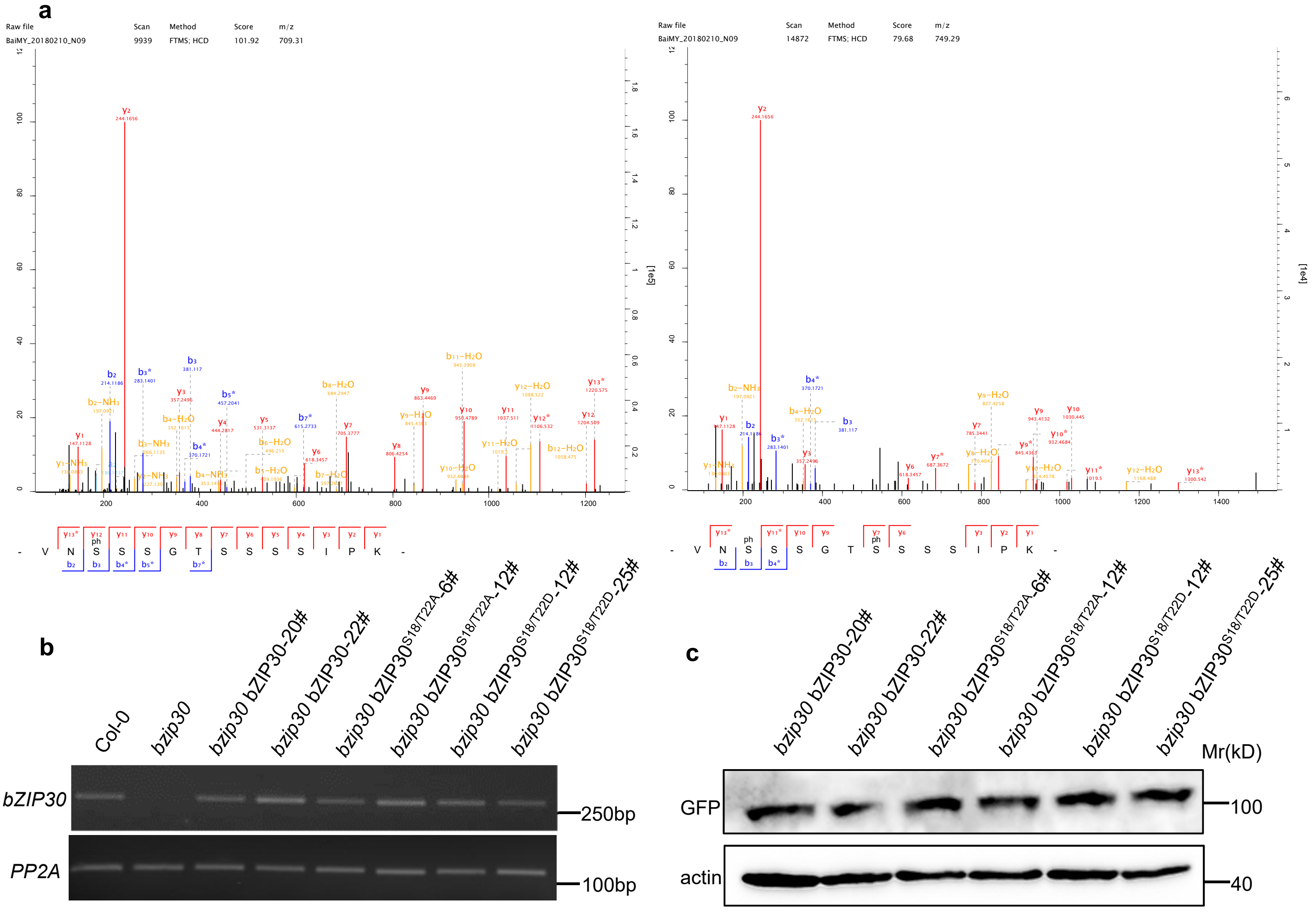

Supplementary Fig. 12 Identification of KIN10 dependent phosphorylation sites on bZIP30. a Mass spectrometry analysis of KIN10 phosphorylation sites on bZIP30. **b** RT-PCR showed the expression levels of *bZIP30* and mutation forms of *bZIP30* in wild type, *bzip30* and different transgenic plants. *PP2A* was used to verfy the equal cDNA loading**. c** Immunoblot analysis showed the protein levels of bZIP30-YFP and mutation forms of bZIP30-YFP in wild type, *bzip30* and different transgenic plants. Actin was used as protein loading control.

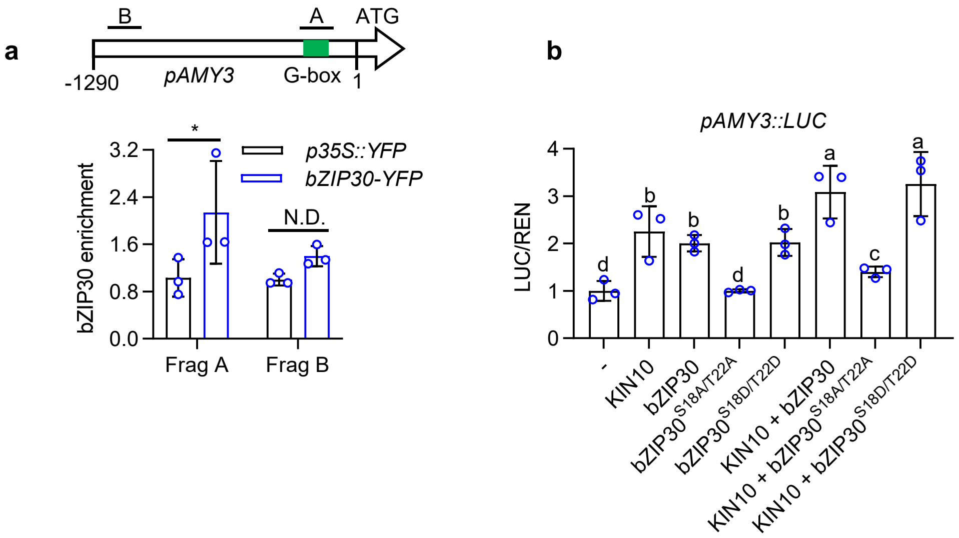

Supplementary Fig. 13 bZIP30 directly induces *AMY3* expression.

a Quantitative ChIP-PCR showed that the direct binding of bZIP30 to *AMY3* promoter. Seedlings of *p35S::YFP* and *pbZIP30::bZIP30-YFP* were used to performed ChIP assays. The levels of bZIP30 binding were calculated as the ratio between *pbZIP30::bZIP30-YFP* and *p35S::YFP*, and then normalized to that of control gene *PP2A*. Error bars indicate standard deviation (S.D.). Asterisks between bars indicate statistically significant differences between samples (Two-way ANOVA analysis followed by uncorrected Fisher's LSD multiple comparisons test, $p < 0.05$). **b** Transient assays showed that the expression of *AMY3* was induced by KIN10-YFP, bZIP30-YFP and bZIP30$^{S18/T22D}$-YFP, but not by bZIP30$^{S18/T22A}$-YFP. The promoter of *AMY3* fused to the luciferase reporter gene was co-transfected with KIN10-YFP, bZIP30-YFP, bZIP30$^{S18/T22A}$-YFP or bZIP30$^{S18/T22D}$-YFP into mesophyll protoplasts of wild-type plants. The luciferase activities were normalized with *Renilla* luciferase as an internal control. Error bars represent standard deviation (S.D.). Different letters above the bars indicate statistically significant differences between samples (One-way ANOVA analysis followed by uncorrected Fisher's LSD multiple comparisons test, $p < 0.05$).

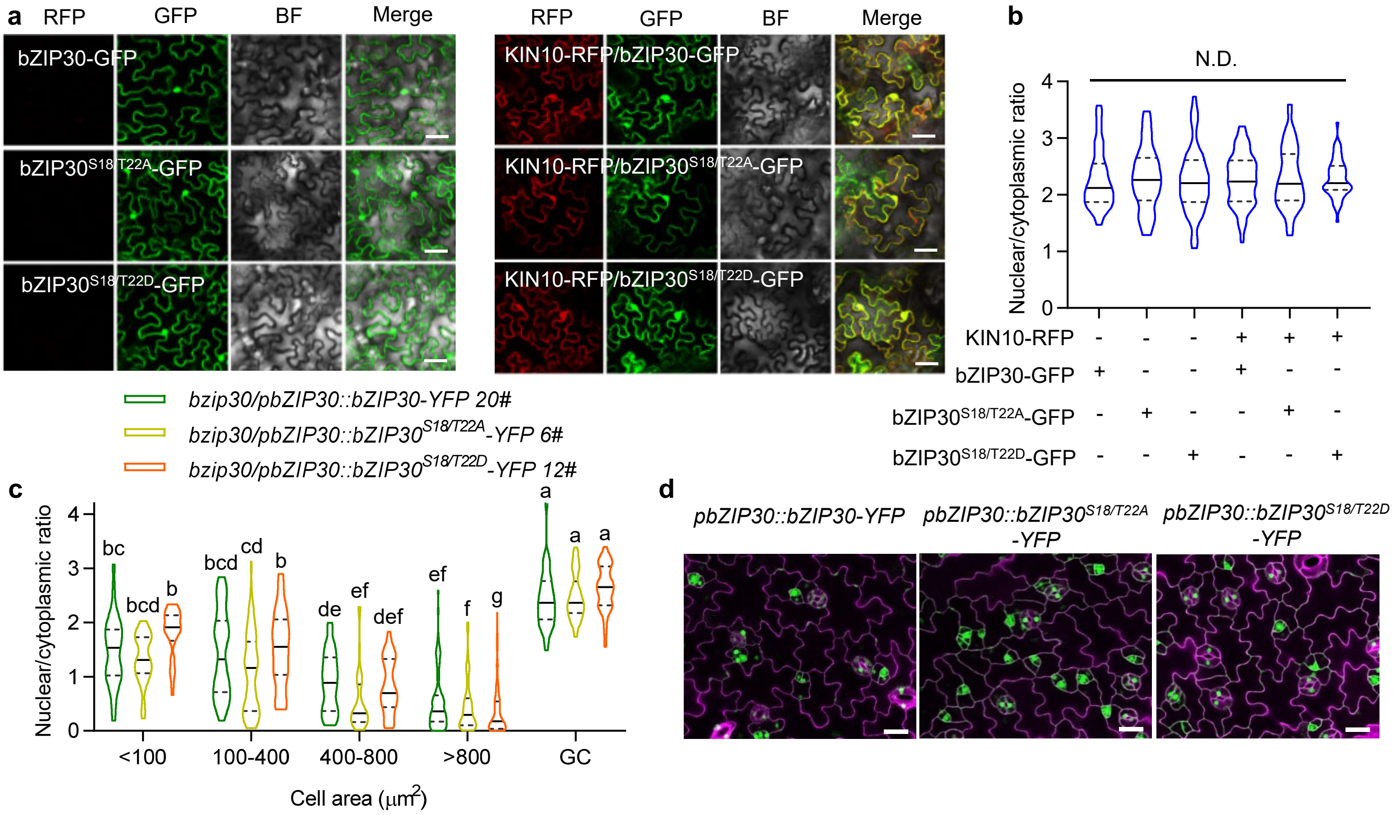

Supplementary Fig. 14 KIN10 dependent phosphorylation did not change bZIP30 subcellular localization. a, b Co-expression of KIN10 did not changes bZIP30 subcellular localization in tobacco leaves. The construct of bZIP30-GFP, bZIP30$^{S18/T22A}$-GFP and bZIP30$^{S18/T22D}$-GFP is transformed alone or co-transformed with KIN10-RFP in tobacco leaves. Nuclear and cytoplasmic fluorescent signals of bZIP30-GFP or mutated bZIP30-GFP from at least 50 epidermal cells were measured using ImageJ. Violin graph (**b**) show the ratio of nuclear and cytoplasmic signals of bZIP30-GFP or mutated bZIP30-GFP. N.D. represent not significant difference between samples (One-way ANOVA analysis followed by uncorrected Fisher's LSD multiple comparisons test). **c, d** Subcellular localization of bZIP30-YFP and mutated bZIP30-YFP in plant. Seedlings of *pbZIP30::bZIP30-YFP*, *pbZIP30::bZIP30$^{S18/T22A}$-YFP* and *pbZIP30:: bZIP30$^{S18/T22D}$-YFP* were grown on 1/2 MS medium under a 12 h light/12 h dark photoperiod with the 100 μM m^{-2} s^{-1} light intensity for 4 days. Violin graph (**c**) show the ratio of nuclear and cytoplasmic signals of bZIP30-YFP or mutated bZIP30-YFP among epidermal cells with different size. Confocal images of bZIP30-YFP or mutated bZIP30-YFP are in green, PI-marked cell outlines are in purple. Scale bars in confocal images represent 20 μm. Different letters above the bars indicate statistically significant differences between samples (Two-way ANOVA analysis followed by Tukey's multiple comparisons test, $p < 0.05$). The solid lines of violin plots in this figure represent median, the dashed lines represent first or third quartile.

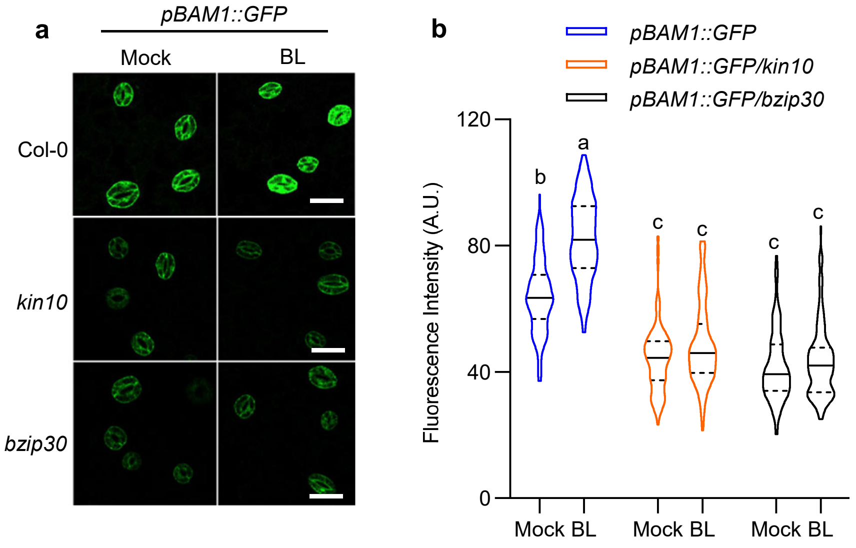

Supplementary Fig. 15 The KIN10-bZIP30 module integrates BR and H_2O_2 signals to induce *BAM1* gene expression. a, b Mutations of KIN10 and bZIP30 inhibit BL induced *BAM1* expression. Transgenic plants *pBAM1::GFP*, *pBAM1::GFP/kin10* and *pBAM1::GFP/bzip30* were grown on 1/2 MS medium under 12 h light/12h dark photoperiod with 100 μM m^{-2} s^{-1} for 10 days, and then treated with or without 100 nM BL for 3 hours. GFP signal from more than 100 guard cells in at least 10 different plants were analyzed by ImageJ software. Scale bars represent 20 μm. The solid lines of violin plots in this figure represent median, the dashed lines represent first or third quartile. Different letters above the bars indicated statistically significant differences between the samples (Two-way analysis ANOVA followed by Tukey's multiple comparisons test, $p < 0.05$). Error bars indicate standard deviation (S.D.).

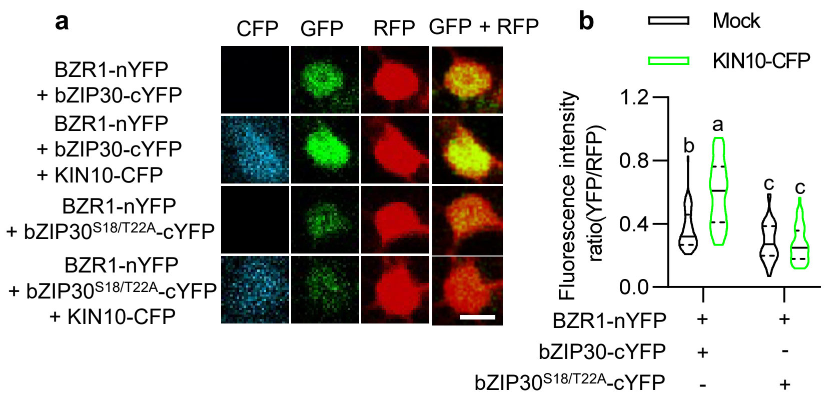

Supplementary Fig. 16 KIN10 dependent phosphorylation on bZIP30 promotes the heterodimerization of bZIP30 and BZR1. **a, b** The rBiFC construct of *p35S::BZR1-nYFP-p35S::RFP-p35S::bZIP30-cYFP* or *p35S::BZR1-nYFP-p35S::RFP-p35S:: bZIP30$^{S18/T22A}$-cYFP* is co-transferred with or without *p35S::KIN10-CFP* in tobacco leaves. The fluorescent signals of YFP (BiFC) and RFP (reference) in the nucleus of tobacco epidermal cells were determined using ImageJ software. At least 50 interaction signals in different epidermal cells were analyzed. The solid lines of violin plots in this figure represent median, the dashed lines represent first or third quartile. Different letters above the bars indicated statistically significant differences between the samples (Two-way ANOVA analysis followed by Tukey's multiple comparisons test, $p < 0.05$. Scale bar,10 μm).

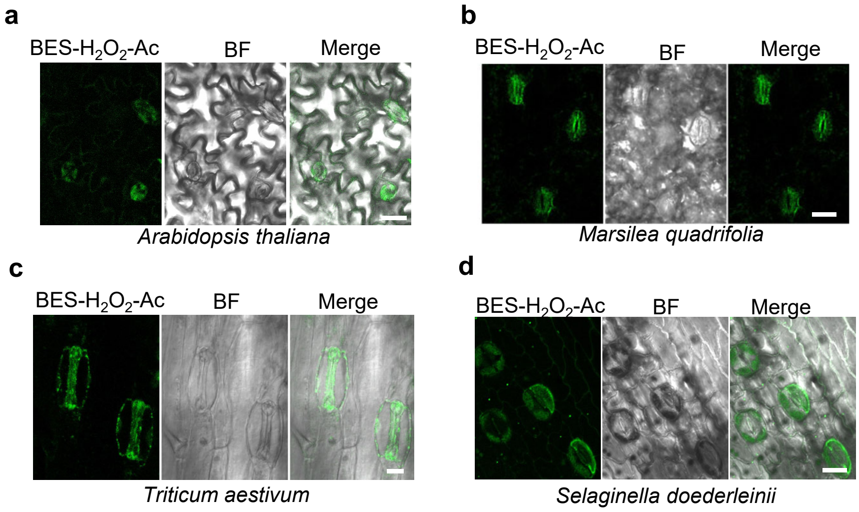

Supplementary Fig. 17 Specific accumulated H_2O_2 in guard cell exists in different plant species. a-d BES-H_2O_2-Ac staining of H_2O_2 in the epidermal cells of *Arabidopsis thaliana* (dicotyledonous, **a**), *Triticum aestivum* (monocotyledonous, **b**), *Marsilea quadrifolia* (ferns, **c**) and *Selaginella doederleinii* (lycophytes, **d**). Scale bars in confocal images for H_2O_2 staining represent 20 μm.

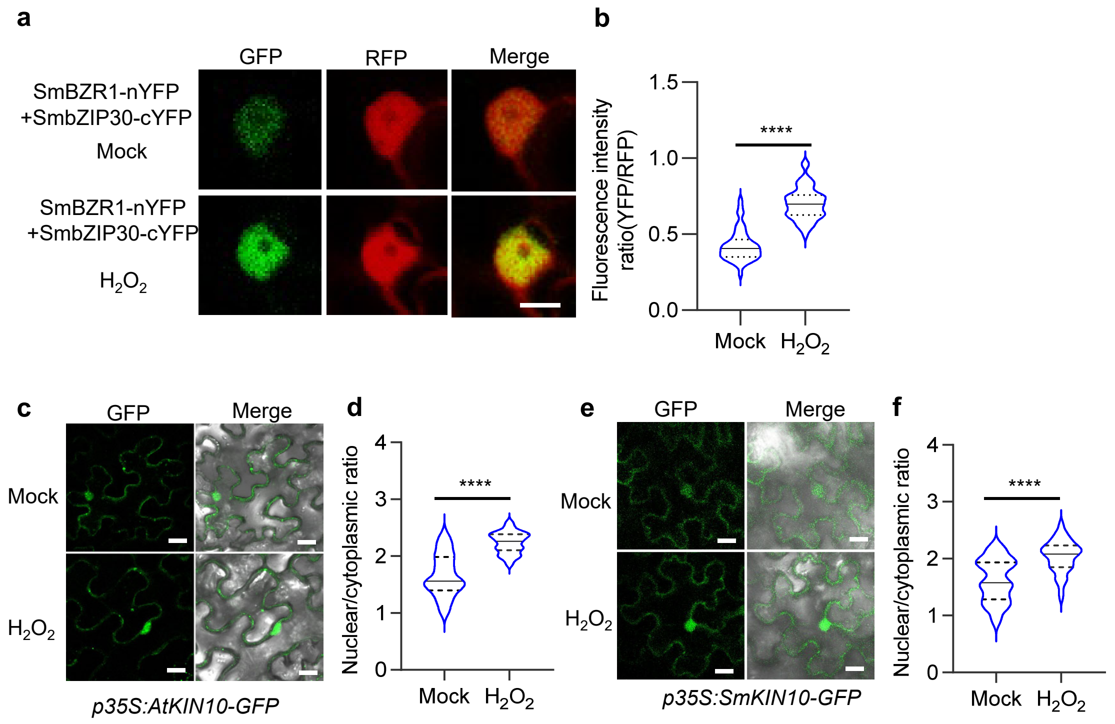

Supplementary Fig. 18 H_2O_2 promotes nuclear localization of KIN10 homolog protein and the heterodimer of bZIP30 and BZR1 homolog protein from *Selaginella moellendorffii*. a, b H_2O_2 promotes SmbZIP30 and SmBZR1 interaction in tobacco leaves. The rBiFC construct of *p35S::SmBZR1-nYFP-p35S::RFP-p35S::SmbZIP30-cYFP* was transferred in tobacco leaves, and treated with 1 mM H_2O_2 for 3 h before observation. The fluorescent signals of YFP (BiFC) and RFP (reference) in the nucleus of tobacco epidermal cells were determined using ImageJ software. At least 50 interaction signals from more than 50 different epidermal cells were analyzed using ImageJ software. The solid lines of violin plots in this figure represent median, the dashed lines represent first or third quartile. Asterisks between bars indicate statistically significant difference between samples (Student t' test, ****$p<0.0001$). Scale bars in confocal images represent 20 μm. **c-f** H_2O_2 promotes AtKIN10 (**c, d**) and SmKIN10 (**e, f**) nuclear localization in tobacco leaves. AtKIN10-GFP and SmKIN10-GFP were transformed in tobacco leaves, then treated with or without 1 mM H_2O_2 for 3 h before observation. Nuclear and cytoplasmic fluorescent signals of AtKIN10-GFP or SmKIN10-GFP from at least 50 epidermal cells were measured using ImageJ. Scale bars in confocal images represent 20 μm.

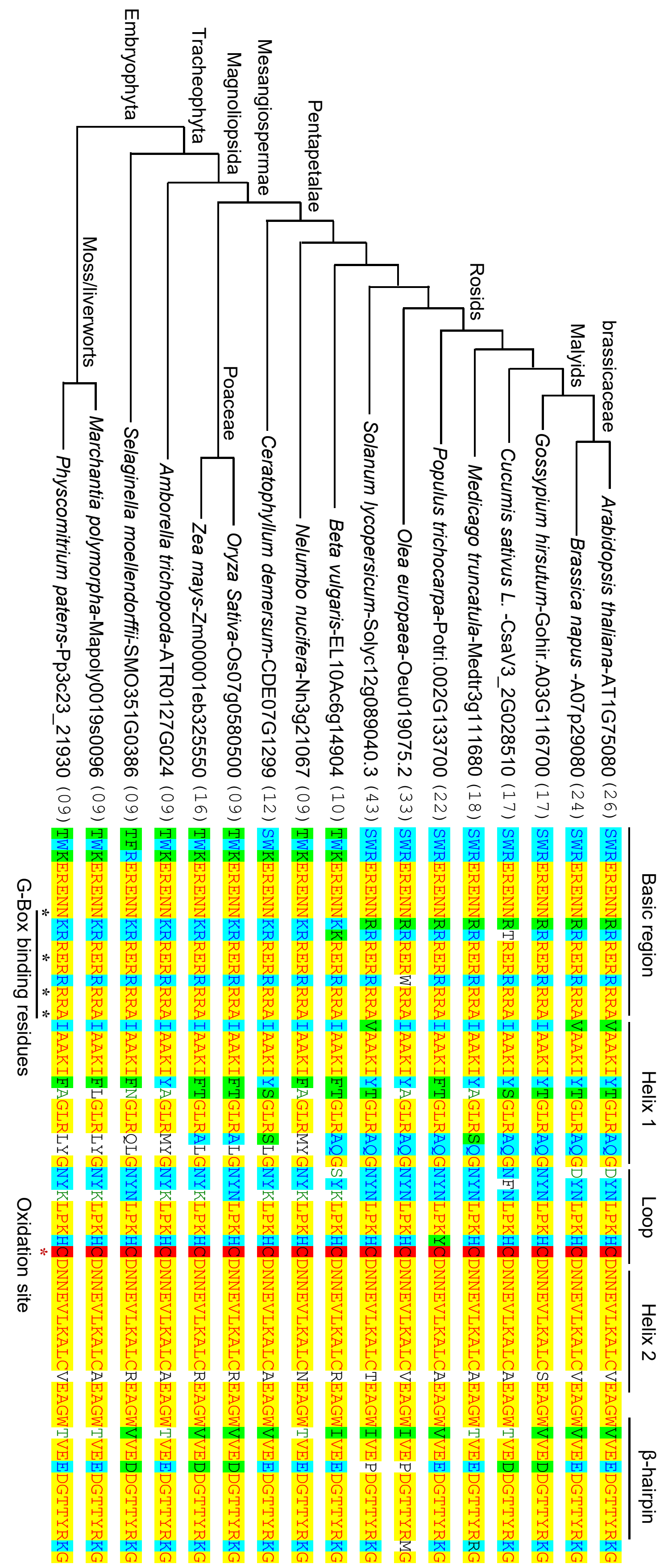

Supplementary Fig. 19 The oxidation site and G-box binding sites in BZR1 orthologs is conserved among land plants. The protein sequences of BZR1 orthologs in represented species of Embryophyta were aligned using ClustalW. The sequences with yellow background represent identical sequences. The sequences with blue background represent conservative sequence. The sequences with green background indicate similar regions. Red background remarks the conserved cysteine of oxidation. The tree represents the most commonly accepted phylogenetic relationships between different species.

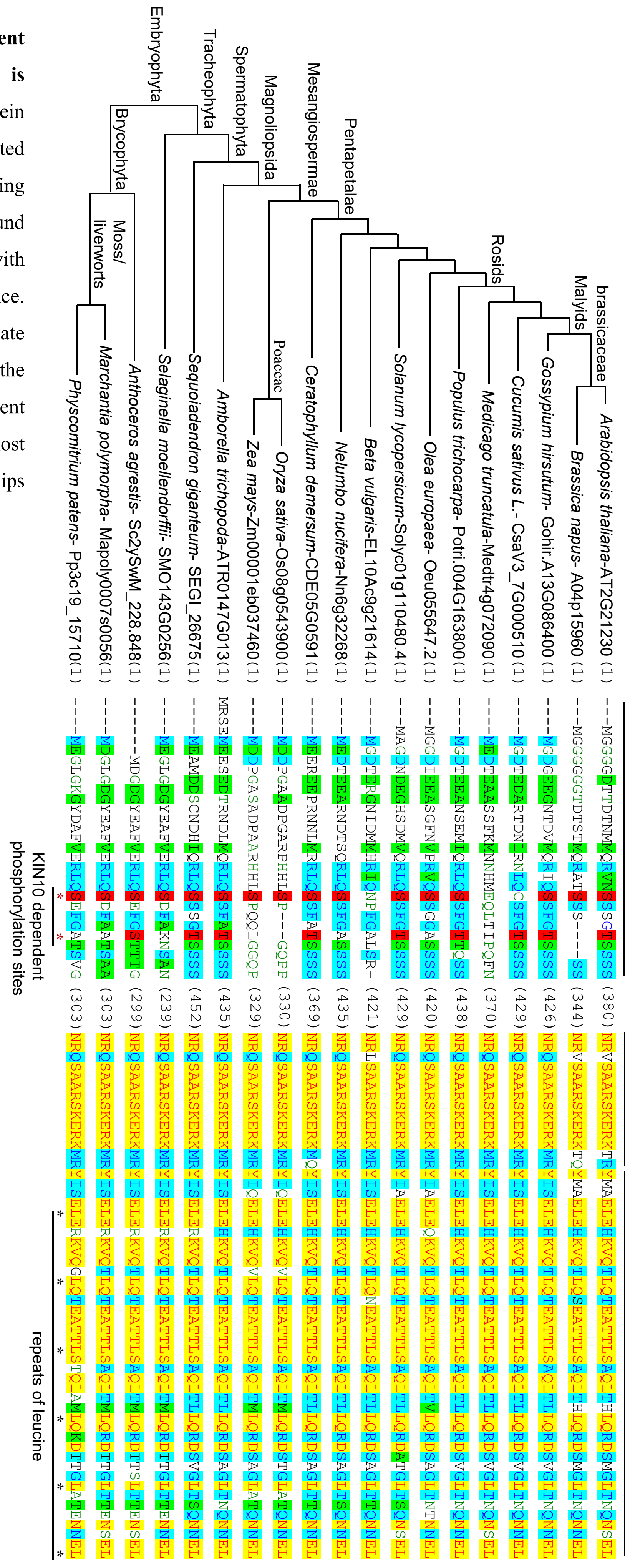

Supplementary Fig. 20 KIN10 dependent phosphorylation sites on bZIP30 orthologs is conserved among land plants. The protein sequences of bZIP30 orthologs in represented species of Embryophyta were aligned using ClustalW. The sequences with yellow background represent identical sequences. The sequences with blue background represent conservative sequence. The sequences with green background indicate similar regions. Red background remarks the serine and threonine of KIN10 dependent phosphorylation. The tree represents the most commonly accepted phylogenetic relationships between different species.

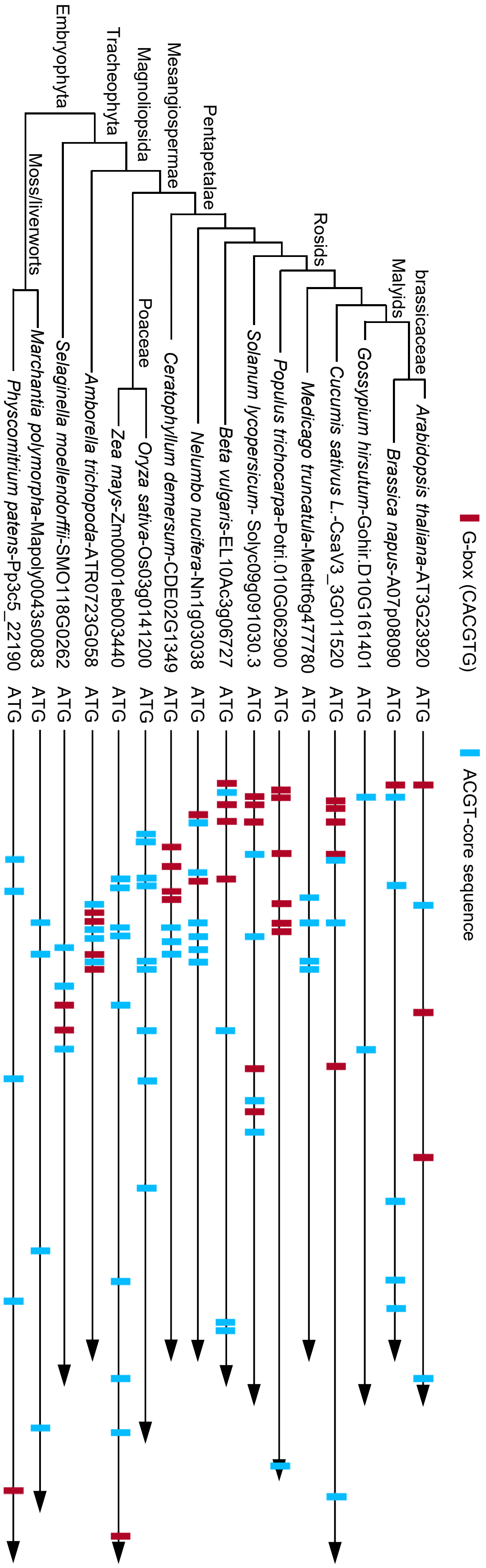

Supplementary Fig. 21 The promoter sequences of *BAM1* ortholog genes contains G-box sequences or core ACGT sequences. The G-box sequences and core ACGT sequences are marked in relative position. The arrows indicate the length of promoters. The promoters contain 5'-UTR and 2000 bp upstream sequences. The tree represents the most commonly accepted phylogenetic relationships between different species.
