# Supplementary material for: Hydrogen peroxide is required for light-induced stomatal opening across different plant species
Source: Nat Commun. 2024 Jun 14;15:5081. doi: 10.1038/s41467-024-49377-9 (PMC11178795; doi:10.1038/s41467-024-49377-9)
Supplement: Supplementary file 3 — Description of Additional Supplementary Files [file 41467_2024_49377_MOESM3_ESM.pdf]

Description of Additional Supplementary Files

Supplementary Data 1 - Oligo primers Used in this Study.

Supplementary Data 2 - Statistical analysis in this study.
