# Supplementary material for: Hydrogen peroxide is required for light-induced stomatal opening across different plant species
Source: Nat Commun. 2024 Jun 14;15:5081. doi: 10.1038/s41467-024-49377-9 (PMC11178795; doi:10.1038/s41467-024-49377-9)
Supplement: Supplementary file 6 — Reporting Summary [file 41467_2024_49377_MOESM6_ESM.pdf]

nature portfolio

Corresponding author(s): Chao Han

Last updated by author(s): May 7, 2024

Reporting Summary

Nature Portfolio wishes to improve the reproducibility of the work that we publish. This form provides structure for consistency and transparency in reporting. For further information on Nature Portfolio policies, see our Editorial Policies and the Editorial Policy Checklist.

Statistics

For all statistical analyses, confirm that the following items are present in the figure legend, table legend, main text, or Methods section.

n/a	Confirmed	
☐	☒	The exact sample size (*n*) for each experimental group/condition, given as a discrete number and unit of measurement
☐	☒	A statement on whether measurements were taken from distinct samples or whether the same sample was measured repeatedly
☐	☒	The statistical test(s) used AND whether they are one- or two-sided<br>*Only common tests should be described solely by name; describe more complex techniques in the Methods section.*
☒	☐	A description of all covariates tested
☒	☐	A description of any assumptions or corrections, such as tests of normality and adjustment for multiple comparisons
☐	☒	A full description of the statistical parameters including central tendency (e.g. means) or other basic estimates (e.g. regression coefficient) AND variation (e.g. standard deviation) or associated estimates of uncertainty (e.g. confidence intervals)
☐	☒	For null hypothesis testing, the test statistic (e.g. *F*, *t*, *r*) with confidence intervals, effect sizes, degrees of freedom and *P* value noted<br>*Give P values as exact values whenever suitable.*
☒	☐	For Bayesian analysis, information on the choice of priors and Markov chain Monte Carlo settings
☒	☐	For hierarchical and complex designs, identification of the appropriate level for tests and full reporting of outcomes
☒	☐	Estimates of effect sizes (e.g. Cohen's *d*, Pearson's *r*), indicating how they were calculated

Our web collection on statistics for biologists contains articles on many of the points above.

Software and code

Policy information about availability of computer code

Data collection	Real-time PCR cycler (CFX96, Bio-Rad) was used for detecting gene expression pattern, and the data were collected by Opticon Monitor. All the experiments were carried out with at least three independent replicates to exclude random effects.
Data analysis	Two-tailed Student's t tests and oneway or two way ANOVA test were conducted using graphpad software. Analysis of Western blot results was performed using ImageJ software (US National Institutes of Health).

For manuscripts utilizing custom algorithms or software that are central to the research but not yet described in published literature, software must be made available to editors and reviewers. We strongly encourage code deposition in a community repository (e.g. GitHub). See the Nature Portfolio guidelines for submitting code & software for further information.

Data

Policy information about availability of data

All manuscripts must include a data availability statement. This statement should provide the following information, where applicable:

- Accession codes, unique identifiers, or web links for publicly available datasets
- A description of any restrictions on data availability
- For clinical datasets or third party data, please ensure that the statement adheres to our policy

All data in this study are available in the manuscript or the Supplementary materials. Source data are provided with this paper.

Research involving human participants, their data, or biological material

Policy information about studies with human participants or human data. See also policy information about sex, gender (identity/presentation), and sexual orientation and race, ethnicity and racism.

Reporting on sex and gender	Our study did not involved in this issue
Reporting on race, ethnicity, or other socially relevant groupings	Our study did not involved in this issue
Population characteristics	Our study did not involved in this issue
Recruitment	Our study did not involved in this issue
Ethics oversight	Our study did not involved in this issue

Note that full information on the approval of the study protocol must also be provided in the manuscript.

Field-specific reporting

Please select the one below that is the best fit for your research. If you are not sure, read the appropriate sections before making your selection.

☒ Life sciences ☐ Behavioural & social sciences ☐ Ecological, evolutionary & environmental sciences

For a reference copy of the document with all sections, see nature.com/documents/nr-reporting-summary-flat.pdf

Life sciences study design

All studies must disclose on these points even when the disclosure is negative.

Sample size	The sample size in this study is mainly determined according to the prior experiences, which are based on the reproducibility and statistical significance of the results during the experiments.
Data exclusions	No data was excluded from the analyses.
Replication	The number of replication are indicated in the figure legends. All experiments were conducted at least three replicates. All data presented are reliable and reproducible.
Randomization	All samples were arranged randomly into experimental groups.
Blinding	All the experiments were performed without prior knowledge of the final outcome, and therefore blinding was not applied.

Reporting for specific materials, systems and methods

We require information from authors about some types of materials, experimental systems and methods used in many studies. Here, indicate whether each material, system or method listed is relevant to your study. If you are not sure if a list item applies to your research, read the appropriate section before selecting a response.

Materials & experimental systems

n/a	Involved in the study	
☐	☒	Antibodies
☒	☐	Eukaryotic cell lines
☒	☐	Palaeontology and archaeology
☒	☐	Animals and other organisms
☒	☐	Clinical data
☒	☐	Dual use research of concern
☐	☒	Plants

Methods

n/a	Involved in the study	
☒	☐	ChIP-seq
☒	☐	Flow cytometry
☒	☐	MRI-based neuroimaging

Antibodies

Antibodies used	anti-GFP (TransGen Biotech, Catalog: HT801-01-100UL, 1:5000 dilution), anti-MYC (Sigma-Aldrich, clone 9E10, Catalog: M4439, 1:5000 dilution), anti-MBP (TransGen Biotech, Catalog: HT701-01-100UL,1:5000 dilution), anti-GST (TransGen Biotech, Catalog:

HT601-01-100UL,1:5000 dilution), anti-Actin (Sigma-Aldrich, Catalog: A0480-200UL, 1:5000 dilution). HRP-conjugated anti-mouse (Catalog:1706516) secondary antibodies were purchased from Bio-Rad. anti-KIN10(Agrisera, Catalog: AS10919, 1:1000 dilution)

Validation

Information of Anti-GFP validation can be found at the product website. <http://www.transgenbiotech.com/index.php/product/index/g/c/id/270.html>
Information of Anti-MYC validation can be found at the product website. <https://www.sigmaaldrich.com/catalog/product/sigma/m4439?lang=zh&region=CN>
Information of Anti-MBP validation can be found at the product website. <http://www.transgenbiotech.com/index.php/product/index/g/c/id/269.html>
Information of Anti-GST validation can be found at the product website. <http://www.transgenbiotech.com/index.php/product/index/g/c/id/268.html>
Information of Anti-actin validation can be found at the product website. <https://www.sigmaaldrich.com/catalog/product/sigma/a0480?lang=zh&region=CN>
Information of secondary antibodies validation can be found at the product website. <http://www.bio-rad.com/zh-cn/sku/1706516-goat-anti-mouse-igg-h-l-hrp-conjugate?ID=1706516>
Information of Anti-KIN10 antibodies validation can be found at the product website. <https://www.agrisera.com/en/artiklar/akin10-snf1-related-protein-kinase-catalytic-subunit-alpha-kin10.html>

Plants

Seed stocks

The T-DNA insertion mutants of bzip30 (SALK_076998), kinβ2-1 (SALK_037416C) and kinβ2-2 (SALK_052521) were ordered from the Arabidopsis Biological Resource Center. cat2, rbohD rbohF and kin10 have been described previously.

Novel plant genotypes

Some novel constructs were introduced into Agrobacterium tumefaciens (strain GV3101) and transformed into bzip30 or kin10 plants by the floral dipping method. We created pbZIP30::bZIP30-YFP/bzip30, pbZIP30::bZIP30S18/T22D-YFP/bzip30, pbZIP30::bZIP30S18/T22A-YFP/bzip30, pKIN10::KIN10-YFP/kin10, pKIN10::NLS-KIN10-YFP/kin10 and pKIN10::NES-KIN10-YFP/kin10 lines.

Authentication

We applied genotype analysis using LB1.3 primer to identify T-DNA insertion.
